# Uterine Foxl2 regulates the adherence of the Trophectoderm cells to the endometrial epithelium

**DOI:** 10.1186/s12958-018-0329-y

**Published:** 2018-02-07

**Authors:** Michal Elbaz, Ron Hadas, Louise M. Bilezikjian, Eran Gershon

**Affiliations:** 1 Department of Ruminant Science, Agricultural Research Organization, 50250 Rishon LeZion, Israel; 2 Department of Biological Regulation, Weizmann Institute of Science, 76100 Rehovot, Israel; 3 Clayton Foundation Laboratories for Peptide Biology and Laboratory of Neuronal Structure and Function, Salk Institute for Biological Studies, La Jolla, San Diego, CA 92037 USA

**Keywords:** FOXL2, Uterine receptivity, FOXL2 depletion, FOXL2 overexpression, Embryo attachment, Gene expression, Cell adherence

## Abstract

**Background:**

Forkhead Transcription Factor L2 (FOXL2) is a member of the forkhead family with important roles in reproduction. Recent studies showed that FOXL2 is expressed in human and bovine endometrium and that its levels fluctuate during pregnancy. In this study, we aimed at evaluating the expression and function of FOXL2 in embryo implantation.

**Methods:**

Mouse uteri at different days of pregnancy were isolated and analyzed for the expression and localization of FOXL2. A lentiviral strategy was further employed to either knockdown or overexpress FOXL2 in non-receptive human endometrial AN3-CA cells and in receptive Ishikawa cells, respectively. These genetically modified cells were compared to cells infected with a control lentivirus to determine the function of FOXL2 in trophectoderm cells adherence to Endometrial Epithelium was associated with the expression of genes known to be involved in acquisition of uterine receptivity.

**Results:**

We report that FOXL2 is expressed in both, the luminal epithelium and the myometrium of the mouse uterus and that its expression declines prior to implantation. We found that endometrial cells expressing low FOXL2 levels, either endogenous or genetically manipulated, were associated with a higher attachment rate of mouse blastocysts or human Jeg3 spheroids and mouse blastocysts. In accordance, low-FOXL2 levels were associated with changes in the expression level of components of the *Wnt/Fzd* and apoptotic pathways, both of which are involved in uterine receptivity. Furthermore, FOXL2 expression was inversely correlated with G-protein signaling protein 2 (*Rgs2*) and cytokine expression.

**Conclusions:**

These results suggest that FOXL2 interferes with embryo attachment. Better understanding of the function of FOXL2 in the uterus would possibly suggest novel strategies for treatment of infertility attributed to repeated implantation failure.

**Electronic supplementary material:**

The online version of this article (10.1186/s12958-018-0329-y) contains supplementary material, which is available to authorized users.

## Background

Forkhead transcription factor L2 (FOXL2) is a member of the forkhead family of transcription factors which plays an indispensable role in differentiation of the embryonic gonads into ovaries [1]. Haplo insufficiency of FOXL2 function, resulting from mutations of the *FOXL2* gene has been shown to cause the blepharophimosis–ptosis–epicanthus inversus syndrome (BPES), a genetic disorder characterized by eyelid and mild craniofacial abnormalities, associated with premature ovarian failure in a subset of affected women [2].

The use of DNA chip and quantitative RT-PCR (qRT-PCR) to detect its potential transcriptional targets in granulosa-like cells, revealed that *Foxl2* affects the expression of genes involved in reactive oxygen species (ROS) detoxification, inflammation and apoptosis [3, 4].

Later studies suggest that *Foxl2* regulates granulosa cell proliferation [5] and ovarian G-protein signaling protein 2 (RGS2) [3, 6]. These multi-functional GTPase-accelerating proteins inactivate the alpha-subunit of G proteins and rapidly switch off the G protein-coupled receptor signaling pathways by promoting GTP hydrolysis [7, 8]. Another recent study found that FOXL2 directly modulates estrogen receptor beta (ESR2) expression through a newly identified intronic element [4]. FOXL2 has also been shown to regulate the expression of follistatin and thereby alters activin and SMAD3 signaling, which are key players in the regulation of reproductive functions by their actions in the ovary and the pituitary [9, 10]. In many species activities of the TGFβ super-family members, including activin-like molecules, play a pivotal role in endometrial remodeling, which is essential for placentogenesis during the peri-implantation period [11]. Interestingly, FOXL2 in the murine pregnant uterus, is exclusively expressed in the implantation sites [7].

The expression of FOXL2 has been shown in human myometrium at term [8]. More recently its expression in human endometrium was reported [12] and its downregulation during the pre-receptive to receptive transition has been described [13]. Another paper demonstrated that FOXL2 expression is downregulated in human endometrial cells upon their co-culture with trophoblast cells [14]. A recent study demonstrated that FOXL2 is expressed in the mouse neonatal mesenchyme and that expression persists in the stroma and the deep inner myometrial layer during uterine maturation [15]. In the adult mouse, FOXL2 is expressed in the differentiated stromal layer [15]. This study further showed that conditional deletion of *Foxl2* in the postnatal uterus results in infertility, reduced thickness of the stroma layer and a hypertrophic, disorganized inner myometrial layer [15]. Furthermore, the supplementary muscular layer fails to form a coherent layer around uterine arteries in mice with postnatal targeted deletion of *Foxl2* [15].

In the present study, we hypothesized that *Foxl2* might play a role in uterus remodeling, preparing the uterine wall for implantation. To challenge this hypothesis we aimed at evaluating the expression and exploring its specific function in regulating critical processes associated with embryo implantation, such as uterine cell proliferation, genes that are involved in apoptosis, and genes that are involved in embryo-maternal recognition, such as *Rgs2* transcript. Our experiments demonstrated that that FOXL2 expression in the mouse uterus is modified along pregnancy. Its significant decline towards implantation is consistent with our findings that endometrial FOXL2 levels inversely correlate with the rate of embryo attachment. These findings go along with the effects of FOXL2 on the expression of genes implicated in uterine maturation and embryo attachment.

## Methods

### Animals

To examine FOXL2 expression during pregnancy, sexually mature, cycling female C57BL/6 mice (7–9 wk. old) were purchased from Harlan (Harlan Laboratories, Rehovot, Israel). The females were mated with C57BL/6 male. The next morning, the females were monitored for vaginal plug (indicating day 0.5 of pregnancy). Uteri were isolated at days 3, 4, 6, 12 and 18 of pregnancy and further analyzed.

In all experiments, three independent repeats were performed as follows: for each time point, uteri, implantation sites and placentas from 3 different random animals were collected. Then, the uteri, placenta or implantation sites collected from the 3 animals at each time point were pooled together and the pool was subjected to further analysis. Thus, a total of 9 uteri/implantation sites/placenta from 9 different random animals were used for each lane.

### Cells

The AN3-CA cells, non-receptive human endometrial cells (cell line obtained from CLS Cell Line Services GmbH, Eppelheim, Germany), were grown in MEM (Biological Industries, Israel) with 10% fetal bovine serum (FBS, Hyclone, Biological Industries, Israel), Ishikawa cells, receptive human endometrial cells (cell line obtained from ATCC, Manassas, VA), were grown in DMEM (Biological Industries, Israel) with 10% FBS. Both cell lines were grown at 37 °C under 5% CO2.

### shRNA-mediated knockdown of FOXL2

The strategy used to achieve siRNA-mediated *Foxl2* knockdown was essentially as described previously [9, 16]. Briefly, lentiviral particles harboring *Foxl2*-targeted shRNA cassettes or scrambled control shRNA in tandem with IRES-controlled GFP cDNA were prepared using HEK293T as described previously [16] and used to infect AN3-CA cells. The cells were expanded under normal growth conditions and monitored for GFP expression. To achieve uniformity of knockdown for functional studies, approximately 10^7^ cells expressing either GFP alone, scrambled shRNA as control or *Foxl2* shRNA were subjected to fluorescence-activated cell sorting (FACS) on a FACSVantage SE DiVa (BD Biosciences, San Jose, CA) equipped with a 488-nm argon laser. Initial gating was based on forward scatter and side scatter to maximize recovery of live single cells. According to the fluorescence intensity histogram of each population of infected AN3-CA cells, the top 5% of GFP+ cells were sorted and collected and expanded for further analysis.

### Lentivirus mediated method of FOXL2 overexpression

Viral particules harboring *Foxl2* cDNA for overexpression or control vector were prepared using HEK293T as described previously [9]. Briefly, Ishikawa cells were infected with control or FOXL2 overexpressing lentivirus that incorporates IRES-driven GFP as a marker. To achieve uniformity of overexpression for functional studies, the transduced Ishikawa cells were FACS sorted as described above and the top 5% of GFP+ cells were collected and expanded for further analysis.

### Establishing a spheroids-endometrial cell attachment assay

The in vitro model for implantation was performed as described previously [17]. Briefly, Jeg3 cells, a human trophoblast cell line, were cultured in a humid atmosphere containing 5% CO_2_ at 37 °C on a shaker for 24 h. The resulting spheres were stained using calcein-AM (BD Bioscience, San Jose, CA), and were monitored using G-BOX gel-imager (syngene, Cambridge, UK), in order to test their viability and to count them. Then, labeled Jeg3 spheroids of 50–200 μm in diameter, similar in size to an implanting blastocyst, were transferred to the upper surface of the confluent monolayer of Ishikawa or AN3-CA endometrial cells, with or without *Foxl2* overexpression or knockdown (approximately 50 spheroids/well), and the co-cultures were maintained for 6 h at 37 °C. At the end of incubation, non-adherent spheroids were removed by washing the culture plates and the plates were examined using a G-BOX gel-imager (syngene, Cambridge, UK). The number of tightly attached spheroids in each well was counted using the GeneTools software. The percentage of attached spheroids relative to the total number of spheroids used to initiate the co-incubation experiments (adhesion percent) was calculated.

### Embryo attachment assay

Embryo attachment assay was performed as described previously [18]. Briefly, Wild-type ICR females were purchased from Harlan and super-ovulated by sub-cutaneous injections of 5 units of pregnant mare’s serum gonadotropin (PMSG, Chrono-gest Intervest, The Netherlands) followed by 5 units of intraperitoneal injection of human chorionic gonadotropin (hCG, Chrono-gest Intervest, The Netherlands) 48 h later and then mated with wild-type ICR males. Morula and blastocyst stage embryos were collected from the females at 3.5 d after copulation and then incubated in KSOM medium to obtain expanded blastocysts. Embryos were labeled with Vybrant Cell-Labeling Solution (ThermoFisher Scientific, Waltham, MS) before transferring unselectively to confluent Ishikawa (infected with control or *Foxl2* overexpression virus or AN3-CA cells infected with control or *Foxl2* siRNA virus) cell monolayers in a 96-well plate coated with Matrigel (In vitro technologies, Victoria, Australia). Between 3 and 4 blastocysts were transferred per well depending upon the total number recovered. Co-cultures were incubated undisturbed at 37 °C in a 5% CO2 atmosphere for 48 h. The stability of embryo attachment was measured by washing the culture plates and shaking them three times from side to side. Each measurement was performed manually under a microscope (Nikon, Tokyo, Japan), by examining the stability of each mouse embryo upon tapping the stage. The percentage of attached embryos relative to the total number of embryos used to initiate the co-incubation experiments (adhesion percent) was calculated (Additional file 1: Figure S1).

### Protein extraction and western blot analysis

Proteins from uteri were extracted at the indicated time points in RIPA buffer using homogenizer, and suspended in Laemmli loading buffer (125 mM Tris, pH 6.8, 4% SDS, 10% glycerol, 0.006% bromphenol blue, and 2% β-mercaptoethanol). The proteins were then separated on a 12% acrylamide gel, followed by their transfer to a nitrocellulose membrane. After blocking with 5% skimmed milk, the membranes were incubated with primary antibodies over-night at 4 °C (rabbit anti-FOXL2 1:1000, Bioss, Woburn, MA; rabbit anti-β-ACTIN 1:2000, Thermo Scientific, Waltham, MS), then with the secondary antibodies for 1 h at room temperature (anti-rabbit 1:5000, Jackson laboratory, Bar Harbor, MI). The immunoreactive bands were detected by ECL (Amersham, England).

### RNA extraction and analysis by PCR

Total RNA from uteri and endometrial cell lines was extracted using RNeasy mini columns (Qiagen, Hilden, Germany), according to the manufacturer’s guidelines. RNA was converted into cDNA with the High-Capacity cDNA Reverse transcription kit (Applied Biosystems), according to the manufacturer’s guidelines using oligo (dT) and Moloney murine leukemia virus reverse transcriptase. The cDNAs were used for PCR amplification with primer sets for *Foxl2* and *Hprt* (Additional file 2: Table S1) in a 25 μl reaction volume, with 10× buffer, 400 μM of each d-NTP and 0.625 units of Taq DNA Polymerase (Fisher Scientific, Waltham, MA). PCR was performed for the indicated number of cycles (initial denaturation at 94 °C for 3 min, then 35 cycles at 94 °C for 1 min, 60 °C for 1 min, 72 °C for 1 min, and a final incubation at 72 °C for 7 min). The reaction mix (10 μl) was run on 1.5% agarose gels, stained with Ethidium Bromide and quantified using UV imaging (Gel Doc 1000, Bio-Rad) and Molecular Analyst software (Bio-Rad, Hercules, Ca.). Experimental replication of each time point was performed in triplicate.

### Quantitive RT-PCR

All real-time PCRs were carried out on a step one plus (ThermoFisher Scientific, Waltham, MS), using the Absolute Blue QPCR Master Mix (ThermoFisher Scientific, Waltham, MS) with SYBR Green. The following is the reaction protocol: 15 min at 95 °C for enzyme activation, followed by 40 cycles of: 15 s at 95 °C, 30 s at 60 °C, and 15 s at 72 °C, at the end of which fluorescence was measured with the Rotor-Gene. SYBR Green-I assays also included a melt curve at the end of the cycling protocol, with continuous fluorescence measurement from 65 to 99 °C. All reactions contained the same amount of cDNA, 10 μl Absolute Blue QPCR Master Mix, primers for the indicated genes (Additional file 2: Table S1) and UltraPure PCR-grade water (Biological Industries, Israel) to a final volume of 20 μl. Each real-time PCR included a no-template control, in duplicate. Relative expression levels (ΔΔCt) were calculated by normalizing to hypoxanthine guanine phosphoribosyl transferase (HPRT). Primers were designed using the primer3 website (http://bioinfo.ut.ee/primer3-0.4.0/).

### Immunofluorescence staining

FOXL2 immunofluorescence was performed on deparaffinized uterine sections isolated from non-pregnant C57/BL6 females. The sections were washed in PBS (Biological Industries, Israel), followed by antigen retrieval by standard sodium citrate method. Non-specific binding sites were blocked by incubating the sections for 30 min in 20% fetal bovine serum (Biological Industries, Israel), 0.2% Triton X100 (Sigma, Rehovot, Israel) in PBS. Sections were then incubated overnight at 4 °C with anti-FOXL2 antibody (anti-FOXL2 rabbit polyclonal antibody, 1:50, Biosis, Woburn, MA). Sections were washed with PBS and immunoreacted with Cy3-conjugated anti-rabbit IgG in 2% normal horse serum and PBS for 60 min (dilution 1:150, Jackson Laboratories, Bar Harbor, MI). The sections were subsequently washed with PBS and visualized, using fluorescence microscope (Nikon, Tokyo, Japan). All images were taken in identical conditions.

### Statistical analysis

For statistical comparisons, replicate experiments were averaged and using Student’s 2 tailed unpaired *t*-test and considered statistically different when *P* < 0.05. These statistical analyses were conducted with JMP software (SAS Institute, 2005). All numerical data shown in the figures are from representative experiments expressed as the means −/+S.E.M of replicates.

## Results

### FOXL2 expression and its localization in the mouse uterus

**Fig. 1:**
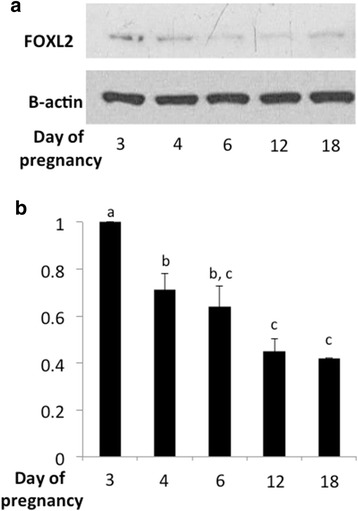
FOXL2 expression in the murine uterus during pregnancy. **a** FOXL2 protein levels decrease prior to implantation (implantation occurs at pregnancy day 4.5). Western blot analysis of FOXL2 protein using β-actin as a loading control. The results represent one out of 3 independent experiments with similar results. **b** Quantitation of three independent experiments with similar results. Different letters represent significant differences (*p* < 0.05)

Our initial experiments were directed at the analysis of the spatio-temporal expression of FOXL2 in the mouse uterus during pregnancy. We found that the FOXL2 protein, which is high upon establishment of pregnancy (days 3 and 4, vaginal plug is day 0.5), significantly decreases prior to implantation (occurring on pregnancy day 4.5 (implantation occurs at day 4.5, vaginal plug is day 0.5, Fig. 1a-b).

**Fig. 2:**
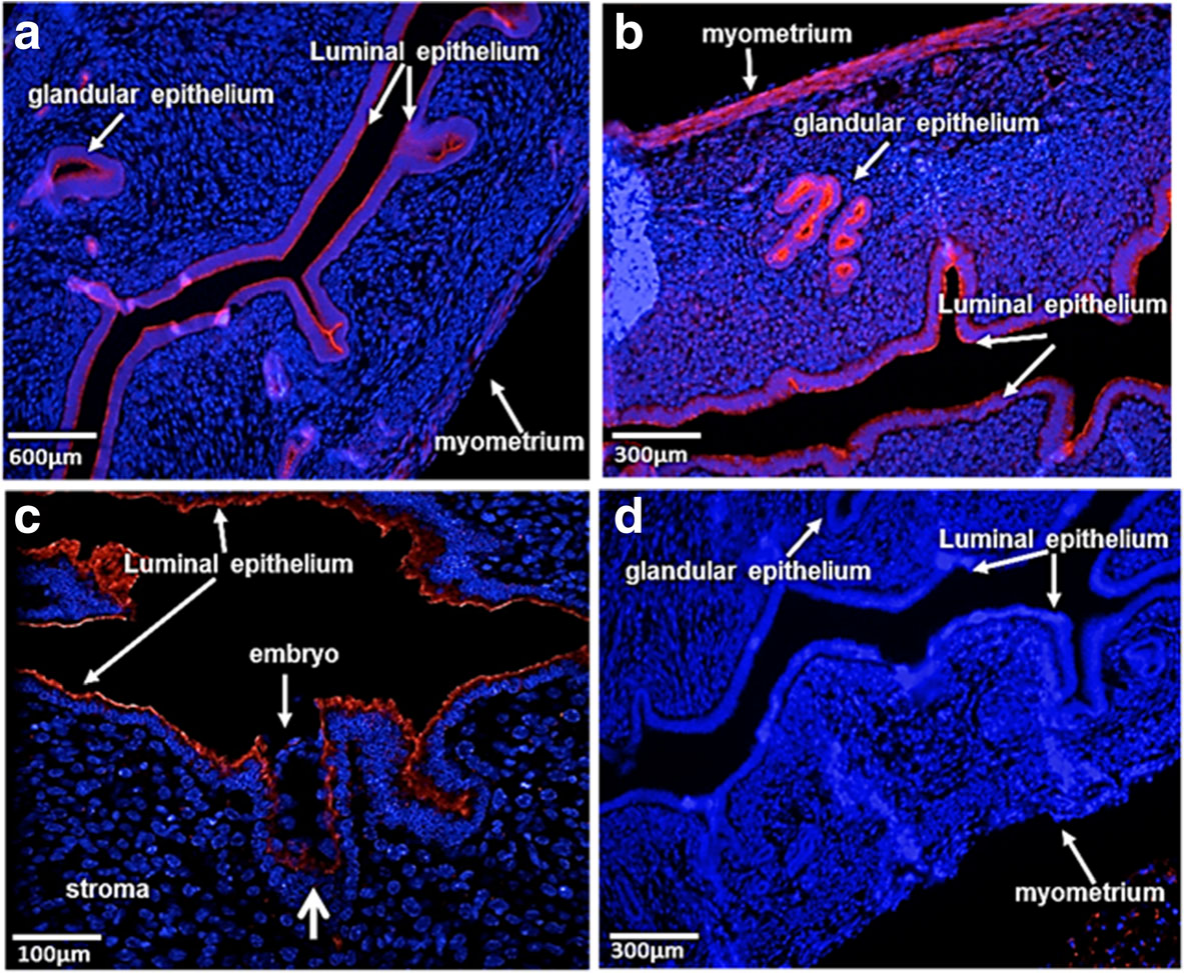
FOXL2 Localization in the Uterus. **a** FOXL2 is localized to the luminal epithelium, myometrium and glandular epithelium in the non-pregnant uterus of a female mouse. **b** A closer view of FOXL2 localization in the non-pregnant uterus. **c** FOXL2 is localized to the luminal epithelium, and to the embryo on day E4.5 of pregnancy. The thick arrow indicates reduced expression of FOXL2 in uterine cells surrounding the attached embryo. **d** No staining is detected in sections incubated without the primary antibody, anti-FOXL2. Blue = DNA staining (DAPI), RED = FOXL2

In the bovine, FOXL2 has been localized to uterine endometrial, stromal and glandular cells [19]. In agreement, using anti-FOXL2, our immunofluorescence analysis of non-pregnant murine uterine sections showed that FOXL2 localizes to the myometrium, glandular epithelium and the luminal epithelium of (Fig. 2a-b). Analysis of pregnant mouse uterine tissue at embryonic day 4.5 revealed that FOXL2 is expressed by the luminal epithelium as well as by the embryo (Fig. 2c). Interestingly, this experiment showed that FOXL2 could also be detected at the attachment area of the embryo with the luminal epithelium. However, the expression of this protein in luminal epithelial cells surrounding the attached blastocysts is substantially lower than that in the luminal epithelial cells away from the implantation site (Fig. 2c). The specificity of the FOXL2 antibody was confirmed by the absence of signal in sections incubated without the primary anti-FOXL2 antibody (Fig. 2d) The decrease in FOXL2 expression in granulosa cells as folliculogenesis progresses (Additional file 3: Figure S2) confirmed the findings in a previous study [20] and was used as a complementary experiment for specificity demonstration.

Analysis of uterine tissue from healthy women showed that FOXL2 is present in human endometrial tissues [12]. We analyzed two human endometrial cell lines, AN3-CA and Ishikawa cells, and confirmed by RT-PCR that both lines express *FOXL2* mRNA (Additional file 4: Figure S3A).

### Establishment of FOXL2 depleted or FOXL2 overexpressing cells

**Fig. 3:**
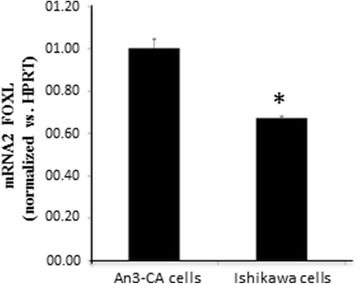
*Foxl2* expression in uterine cell lines. qRT-PCR of *Foxl2* mRNA expression in AN3-CA and Ishikawa endometrial cell lines. Endogenous expression in Ishikawa cells is significantly lower

Our next experiments were directed at the validation of human endometrial cell lines models to be employed for further evaluation of the function of FOXL2 in embryo implantation. We used two human endometrial cell lines, AN3-CA and Ishikawa cells that are considered to be representative of non-receptive and receptive endometrium, respectively ([21, 22] and our unpublished data). Interestingly, the two cell lines differ by the level of endogenous FOXL2, with significantly higher levels of *Foxl2* mRNA in AN3-CA non-receptive endometrial cells (Fig. 3 and Additional file 4: Figure S3b). In order to assess the effect of FOXL2 on endometrial functions, we manipulated Foxl2 levels in the two cell lines by knocking down *Foxl2* in AN3-CA cells by shRNA expression using lentiviral particles harboring *Foxl2* -targeted shRNA (shFOXL2) cassettes and, conversely, overexpressing *Foxl2* in Ishikawa cells using lentiviral particles harboring the FOXL2 cDNA. These strategies resulted in a 60–70% reduction of *Foxl2* mRNA levels in AN3-CA cells infected with sh *Foxl2* -encoding lentivirus (Additional file 1: Figure S1A) and as much as a 25-fold increase in *Foxl2* mRNA levels in Ishikawa cells infected with *Foxl2* overexpressing virus, compared to cells infected with a control virus (Additional file 1: Figure S1B). We used these modified and the parent cell lines to examine the effect of *Foxl2* on embryo attachment.

### Attachment of murine embryos and Jeg3 spheroids to human endometrial cells with FOXL2 either depletion or overexpression

**Fig. 4:**
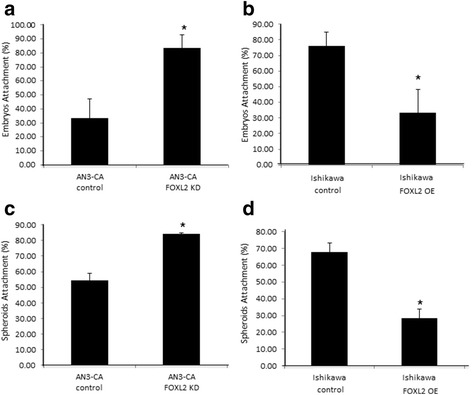
*Foxl2* effects in an in vitro model of attachment. **a** Mouse embryo attachment is higher after *Foxl2* knockdown in AN3-CA cells. **b** Overexpression of *Foxl2* in Ishikawa cells reduces mouse embryo attachment. **c** Jeg3 spheroid attachment is higher after *Foxl2* knockdown in AN3-CA. **d** Overexpression of *Foxl2* in Ishikawa cells decreases Jeg3 spheroid attachment. * *p* < 0.05

The effect of changes in *Foxl2* abundance on embryo attachment was assessed by an in vitro adhesion assay (Additional file 5: Figure S4A-C, [18]). We found that the rate of embryo attachment to *Foxl2* -depleted AN3-CA was higher than that to control AN3-CA cells (Fig. 4a). By contrast, overexpression of *Foxl2* in Ishikawa cells led to a reduction in embryo attachment compared to control Ishikawa cells (Fig. 4b). In a complementary experiment we used the in vitro implantation assay to examine Jeg3 human spheroid attachment to the same endometrial cell lines with either high or low FOXL2 protein expression. Similar to our results with mouse embryo attachment, we observed that, Jeg3 spheroid attachment to *Foxl2* -depleted AN3-CA cells was higher than that to control AN3-CA cells (Fig. 4c). Conversely, overexpression of *Foxl2* in Ishikawa cells led to inhibition of spheroid attachment compared to control Ishikawa cells with lower FOXL2 levels (Fig. 4d). These observations support the possibility that the reduced FOXL2 protein expression seen around the time of implantation (Fig. 1b) contribute to the process of implantation by allowing the attachment of the embryo to the endometrial epithelium.

### FOXL2 effects on the expression of members of the wnt/fzd family

**Fig. 5:**
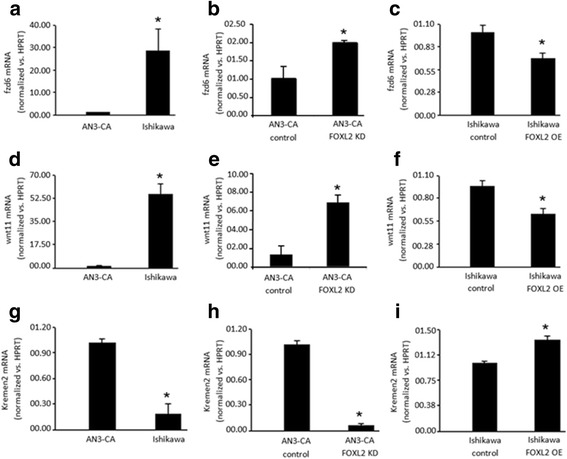
*Foxl2* effect on the expression of Wnt/Fzd family members. **a**, **d**, **g** The expression of *Fzd6* and *Wnt11* is higher, while the expression of *Kremen2*, a Wnt/Fzd family inhibitor, is lower in receptive endometrial Ishikawa cells, which express low *Foxl2* levels, compared to AN3-CA endometrial non-receptive cells, which express higher levels of *Foxl2*. **b**, **e**, **h** Knockdown of *Foxl2* in AN3-CA leads to an increase in *Fzd6* and *Wnt11* expression and a reduction in *Kremen2* levels. **c**, **f**, **i** Overexpression of *Foxl2* in Ishikawa cells leads to a decrease in *Fzd6* and *Wnt11* levels but elevates *Kremen2* levels. * *p* < 0.05

The Wnt/Fzd signaling pathway has been shown to play a role in embryo implantation and stromal cell differentiation [23]. We therefore evaluated the influence of FOXL2 on the expression levels of representative members of the Wnt/Fzd family, *Wnt11* and *Fzd6*, shown to be expressed in the mouse uterus before and around the time of implantation [24]. Using the cellular models described above (Fig. 3 and Additional file 1: Figure S1), we show that *Foxl2* levels are inversely correlated with the expression levels of *Fzd6* and *Wnt11* mRNA. We found that both *Fzd6* and *Wnt11* mRNA are expressed in significantly lower levels in AN3-CA, which contain higher endogenous *Foxl2*, than in Ishikawa cells, which express lower *Foxl2* (Fig. 5a and d). Knockdown of *Foxl2* by siRNAs in AN3-CA cells led to an increase in both, *Fzd6* mRNA (Fig. 5b) and *Wnt11* mRNA (Fig. 5e), while overexpression of *Foxl2* in Ishikawa cells led to a reduction in *Fzd6* mRNA (Fig. 5c) as well as *Wnt11*mRNA (Fig. 5f) expression. We also determined the effect of *Foxl2* levels on the expression of antagonists of this pathway. Indeed, these experiments revealed an opposite effect of FOXL2 on the expression *Kremen2* mRNA, a coreceptor for *Dickkopf 1* and 2, which are Wnt/Fzd signaling antagonists. Cells with lower *Foxl2* expression, either endogenous (Fig. 5g) or genetically manipulated (Fig. 5h), had exhibited lower *Kremen2* mRNA levels (Fig. 5g) whereas FOXL2 overexpression led to induction of *Kremen2* mRNA (Fig. 5i).

### FOXL2 regulation of genes involved in cellular stress responses and apoptosis

**Fig. 6:**
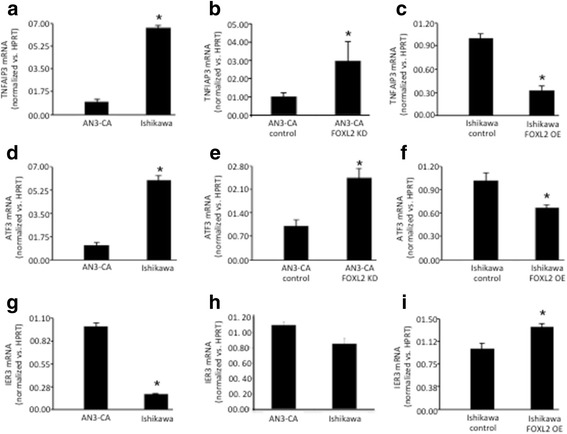
*Foxl2* effect on the expression of genes involved in apoptosis. **a**, **d**, **g** The expression of *Tnfip3* and *Atf3* is elevated, while the expression of *Ier3* is decreased, in receptive endometrial Ishikawa cells, expressing low *Foxl2* levels as compared to AN3-CA, endometrial non-receptive cells, expressing high levels of *Foxl2*. **b**, **e**, **h** *Tnfip3* and *Atf3* expression is reduced, while the expression of *Ier3* is elevated in AN3-CA after *Foxl2* knockdown. **c**, **f**, **i** Overexpression of *Foxl2* in Ishikawa cells is associated with a decrease in *Tnfip3* and *Atf3* levels and increase in *Ier3* levels. * *p* < 0.05

Uterine cell apoptosis is an important process for normal uterine function [25]. FOXL2 has been implicated in the regulation of processes such as apoptosis, by affecting the expression of relevant genes, including IER3, TNFAIP3 and ATF3 [3]. We evaluated if FOXL2 is also involved in the regulation of these genes in the endometrium. We found that *TNFAIP3* mRNA (Fig. 6a and b) and *ATF3* mRNA (Fig. 6d and e) are expressed at higher levels in cells with lower levels FOXL2, namely Ishikawa cells or AN3-CA cells following FOXL2 knockdown. Overexpression of FOXL2 in Ishikawa cells, on the other hand, was associated with a reduction in *TNFAIP3* and *ATF3* mRNA levels (Fig. 6c and f). By contrast, *IER3* mRNA levels were lower in cells with low endogenous FOXL2 (Ishikawa cells) and induced upon FOXL2 overexpression (Fig. 6g and i). AN3-CA cells with FOXL2 depletion, exhibit tendency to express lower levels of *IER3* (Fig. 6h).

### FOXL2 effects on genes involved in embryo implantation and uterus receptivity

**Fig. 7:**
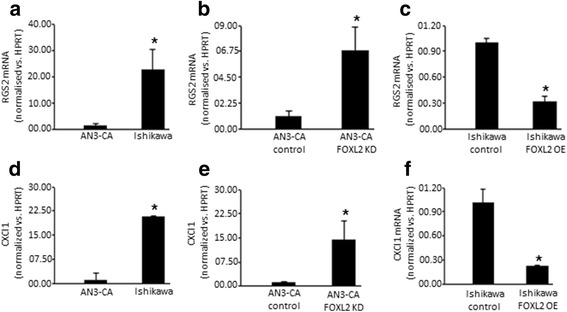
*Foxl2* effect on the expression of genes involved in embryo implantation. The expression of *Rgs2* and *Cxcl1* is elevated in receptive endometrial Ishikawa cells, expressing low *Foxl2* levels as compared to AN3-CA, endometrial non-receptive cells, expressing high levels of *Foxl2*, (**a**, **d**) as well as in AN3-CA *Foxl2* depleted cells (**b**, **e**). Overexpression of *Foxl2* in Ishikawa cells resulted in a decrease in *Rgs2* and *Cxcl1* levels (**c**, **f**). * *p* < 0.05

Uterus receptivity, embryo-maternal recognition and embryo implantation are processes regulated by multiple embryonic and maternal uterine genes. Of these, one of the most intriguing is RGS2, the expression of which in murine pregnant uterus is exclusive to the implantation sites [7]. Another gene that has been demonstrated to play a role in the crosstalk between the embryo and the uterus is the chemokine, CXCl1 [26]. Interestingly, both RGS2 and CXCl1 were reported to be targets of FOXL2 in the ovary [3]. We examined the mRNA expression of both *Rgs2* and *Cxcl1* in our low- and high-FOXL2 expressing cell lines. We found that *Rgs2* mRNA levels are higher in the Ishikawa cells, which express lower levels of FOXL2 and are considered to be representative of a receptive endometrium, as compared to AN3-CA cells, a non-receptive endometrial cell type with higher levels of endogenous FOXL2 (Fig. 7a). Consistent with this trend, *Rgs2* mRNA levels were increased or reduced following FOXL2 knockdown in AN3-CA cells or FOXL2 overexpression in Ishikawa cells, respectively (Fig. 7b and c). Similar to the pattern seen for *Rgs2*, high or low FOXL2 protein expression was inversely associated with low or high *Cxcl1* mRNA expression, respectively (Fig. 7d-f).

## Discussion

We demonstrate in this study that FOXL2 is expressed by both human endometrial cells and mouse uteri, and that FOXL2 protein expression in the mouse uterus is pregnancy-stage dependent. We found that FOXL2 is localized to the luminal and glandular epithelium as well as the myometrium of both, non-pregnant and pregnant female mice. FOXL2 expression has been only recently discovered in mouse, human and bovine uteri [12, 15, 27], thus little is known about its role in this tissue. To unveil the functions of FOXL2 in the uterus, we evaluated its influence on the attachment of mouse embryos and spheroids generated from the human trophoblast cell line JEG3 to endometrial cells expressing either low or high FOXL2 levels. Employing complementary experimental models we show an inverse correlation between the abundance of FOXL2 in the endometrial cells and the success rate of trophectoderm cells adherence to Endometrial Epithelium. Our experiments also reveal negative effects of *Foxl2* on the expression of genes known to play crucial roles in uterine function. Altogether our data suggest that, by controlling the expression profile of endometrial genes, *Foxl2* might have an important role in regulating uterus receptivity and embryo implantation. In support to our findings it has been previously reported that *Foxl2* expression decrease during the pre-receptive to receptive transition as well as after co-culturing of human endometrial cells with trophoblast cells [14].

In the current study, we show that FOXL2 localizes to the luminal epithelium and the myometrium. A recent study of neonatal and adult mouse uteri also found FOXL2 protein in the myometrium, but not in the endometrium [15]. The discrepancy between this study and our results might be due to the different methods used. Bellessort et al. used *FOXL2*^Lacz^ mice while we used immunoflorsence staining with FOXL2 specific antibody. Taken altogether, these studies combined with our data suggest that FOXL2 has a key role in uterus remodeling and towards its preparation for implantation. A study of the expression pattern of *Foxl2* in bovine uterus confirmed its presence in the endometrium and showed that *Foxl2* levels are regulated in a hormonal dependent manner [27]. This study showed that both *Foxl2* transcript and FOXL2 protein were expressed from day 5 to day 20 of the estrous cycle, with significant increases during the luteolytic phase followed by a gradual decline corresponding to increased progesterone levels. Consistent with the latter, progesterone supplementation was found to suppress FOXL2 protein levels [27]. These observations are in line with our findings in mouse uteri and human endometrial cells, suggesting that the roles of *Foxl2* in the uterus might be conserved across species, including humans, mice and farm animals. Further studies are needed to examine this possibility. With respect to this, some differences between mice and goats have been found in *Foxl2* effects on gonadal differentiation and function [6, 20, 28–31].

As mentioned above, *Foxl2* expression has been only recently discovered in human, bovine and murine uteri [12, 15, 19, 27]. Thus, little is known about its role in this tissue. In a recent study [15], conditional deletion of *Foxl2* in the postnatal uterus resulted in infertility, severely reduced thickness of the stroma layer and a hypertrophic, disorganized appearance of the myometrium [15]. Moreover, uterine-specific deletion of *Foxl2* in the adult animal resulted in the appearance of a supplementary muscular layer at the stroma/myometrium border and failure of vascularization of smooth muscle around uterine arteries [15]. In order to expand our understanding of the role of FOXL2 in the human uterus, we utilized two human endometrial cell types to examine the effects of *Foxl2*, either knockdown or overexpression.

In agreement with the findings of Bellassort et al. [15], we observed a reduction in the successful attachment of either mouse blastocysts or Jeg3 trophoblast cells to human Ishikawa receptive endometrial cells with overexpression of *Foxl2*, compared to control Ishikawa cells. Conversely, we found that knockdown of *Foxl2* in AN3-CA non-receptive cells, which express higher levels of endogenous *Foxl2*, improved the attachment of mouse blastocysts and Jeg3 spheroids. Our findings from experiments with human endometrial cells, combined with data from mouse uteri reported by Bellassort et al. [15] support the notion that *Foxl2* plays a negative role in the acquisition uterine receptivity and processes involved in its preparation towards embryo attachment. This notion is further supported by our finding that *Foxl2* is expressed in a lesser extend in uterine epithelial cells surrounding the attached embryo than cells distant from the implantation site.

Additionally, *Wnt* genes were found to be deregulated in this conditional deletion model [15]. Both FOXL2 and the Wnt/Fzd family are necessary for the development of the ovary [32]. In addition, many members of the Wnt/Fzd family are expressed in the uterus [33]. Different Fzd receptors including Fzd6 and Wnt ligands such as Wnt11 were shown to be expressed in the mouse uterus before and around the time of implantation, as well as during stromal cell differentiation [24]. *Wnt11* was detected in the uterus endometrium and epithelial glands in the non-pregnant uterus and its expression was shown to be unregulated by progesterone [24, 34, 35]. *Wnt11* was shown to regulate endometrial gland development [35]. During pregnancy, WNT11 is adjacent to the embryo and FZD6 is localized in the endometrium and stroma during implantation [35].

Our results show an inverse relationship between Wnt/Fzd and *Foxl2* levels. Low *Foxl2* levels are associated with an elevation in *Wnt11* and *Fzd6* and reduction in the levels of Wnt/Fzd inhibitor, *Kremen2*, while the opposite is seen in cells with high *Foxl2* expression levels. Consistent with these findings, conditional deletion of *Foxl2* in the uterus was also found to disrupt *Wnt* gene expression [15]. These results suggest that *Foxl2* has a role in the regulation of normal uterine function, such as uterine glandular generation. Indeed, the decrease in *Foxl2* expression on day 3 of pregnancy might be a crucial mechanism for Wnt/Fzd pathway activation and embryo implantation. Consistent with this notion, the aberrant expression of *Dkk1*, a KREMEN2 receptor, was demonstrated to cause impairment in embryo attachment and implantation [21]. Altogether, these findings provide support for the possibility that conditions that elevate *Foxl2* expression or activity would compromise embryo implantation.

Apoptosis plays an important role in the uterus and is necessary for embryo implantation. *Foxl2* has been implicated in the regulation of both anti- and pro-apoptotic processes in the ovary, based upon microarray data obtained from an ovarian cell line transfected with *Foxl2* [3]. This study reported that *Foxl2* overexpression activated the transcription of several anti-apoptotic genes, such as immediate-early response 3 (*Ier3)*, BCL2-related protein A1 (*Bcl2A1*) and tumor necrosis factor alpha-induced protein 3 (*Tnfaip3*) [3], but also increased transcription of pro-apoptotic factors, including activating transcription factor 3 (*Atf3*) [3]. Our results from endometrial cells suggest that, in parallel to the ovary, FOXL2 affects the expression of both, anti-apoptotic and pro-apoptotic genes. This apparently ambivalent behavior of *Foxl2* on apoptosis is likely to reflect the complexity of the pathways that directly, or indirectly regulate apoptosis and the manner in which differential interactions of *Foxl2* might influence or contribute to processes of uterine cell differentiation, proliferation or programmed cell death. The transcriptional regulation of apoptotic proteins is altered in the endometrium of infertile women with chronic endometriosis as compared to healthy women [36]. Estrogen promotes uterine cell apoptosis [37] and estrogen production and actions are influenced by *Foxl2* [4]. Based upon these observations, we predict that altered *Foxl2* expression or activity would compromise uterine cell apoptosis and ultimately lead to impairment of uterine function and the progression of uterine pathology. This prediction requires further investigations although it is consistent with a recent study showing that *Foxl2* levels are elevated in the endometrium of patients with endometriosis [12].

A variety of factors, including *Rgs2* and chemokines, such as *Cxcl1*, are implicated in mediating the crosstalk between the embryo and the uterus [7, 26]. Both factors were reported to be FOXL2 targets in the ovary [3]. Of these, *Rgs2*, exclusively expressed in the implantation sites of the murine pregnant uterus, is of particular interest [3, 7]. RGS’s are multi-functional, GTPase-accelerating proteins that promote GTP hydrolysis by the alpha-subunit of G proteins, thereby inactivating G proteins and rapidly switching off G protein-coupled receptor signaling pathways. It is thought that RGS2 regulates implantation and pregnancy by influencing intracellular calcium flux or, alternatively, that it participates in the local immunoregulation of uterine tissues during implantation by activating T cells and interleukin-2 production [38] and attenuating the bioactivity of *Mnsfβ*, a molecule implicated in uterine immune tolerance during pregnancy in mice [39]. Similarly, chemokines, such as *Cxcl1*, are implicated in immunoregulatory and inflammatory processes that play a pivotal role in embryo implantation [26]. Here we show that the expression of both *Rgs2* and *Cxcl1* is sensitive to the level of *Foxl2*, suggesting that *Foxl2* might indirectly regulate processes that are controlled by *Rgs2* and *Cxcl1*. Our results suggest that *Foxl2* depletion just prior to embryo implantation might be necessary for preparing the uterus for successful attachment of the embryo to the uterine wall.

## Conclusions

Our data showing a decrease in *Foxl2* levels just before implantation are consistent with the notion that *Foxl2* levels must decline to allow successful embryo attachment, which is an essential prelude for pregnancy progression. This is supported by our observations that *Foxl2* overexpression in endometrial cells reduces embryo and spheroid attachment to receptive cells while attachment is improved by *Foxl2* knockdown in non-receptive cells. Our results further suggest that these effects *Foxl2* are mediated by regulating genes involved in those processes. Further investigations into the contributions of *Foxl2* function to uterus remodeling toward embryo implantation will lead to better understanding of these complex processes. Finding *Foxl2* target genes could also unveil new players in the uterus, both under normal and pathological conditions, and lead to development of new means to improve fertility when implantation fails.

## Additional files

Additional file 1: **Figure S1.** Embryos attachment to endometrial cells. Mouse blastocysts co-incubated with endometrial cell lines for 48 h (A). Attached embryos that stayed on the plate after the plates were washed and shaken (B). Thin arrows indicate attached embryos, thick arrow indicate unattached embryos. (C) The localization of osteopentin, an adhesion molecule, between the embryo and the endometrial cells. Osteopentin-green, DAPI-blue. (TIFF 2025 kb)

Additional file 2: **Table S1.** PCR primers list. (DOCX 15 kb)

Additional file 3: **Figure S2.** FOXL2 localization in the ovary. FOXL2 expressed by granulosa cells of early follicles declines at later stages of folliculogenesis. Blue = DNA staining (DAPI), RED = FOXL2. EA-Early antral, GF-Graffian Folicle. (TIFF 2025 kb)

Additional file 4: **Figure S3.*** Foxl2* expression in human endometrial cell lines. A) Human endometrial cell lines express *Foxl2* mRNA. *Foxl2* mRNA expression in AN3-CA and Ishikawa endometrial cell lines was analyzed by RT-PCR, using *Hprt* mRNA as an internal control. B) *Foxl2* levels are shown as fold mRNA expression, normalized to Hprt. (TIFF 2025 kb)

Additional file 5: **Figure S4.** Manipulating *Foxl2* expression in human endometrial cell lines. A) *Foxl2* expression is decreased in endometrial non-receptive AN3-CA cells infected with lentivirus expressing *Foxl2* siRNA cassette. B) Ishikawa cells infected with lentivirus overexpress *Foxl2* exhibit higher FOXL2 levels as compared to control. The results of one representative out of a total of 3 independent experiments with similar results is presented. + * *p* < 0.05. (TIFF 2025 kb)

## Abbreviations

BPES: Blepharophimosis–ptosis–epicanthus inversus syndrome
FOXL2: Forkhead Transcription Factor L2
hCG: Human chorionic gonadotropin
KD: Knockdown
OE: Overexpression
PMSG: Pregnant mare serum gonadotropin
RGS2: G-protein signaling protein 2
