# Supplementary figures and images for: Uterine Foxl2 regulates the adherence of the Trophectoderm cells to the endometrial epithelium
Source: Reprod Biol Endocrinol. 2018 Feb 7;16:12. doi: 10.1186/s12958-018-0329-y (PMC5804001; doi:10.1186/s12958-018-0329-y)

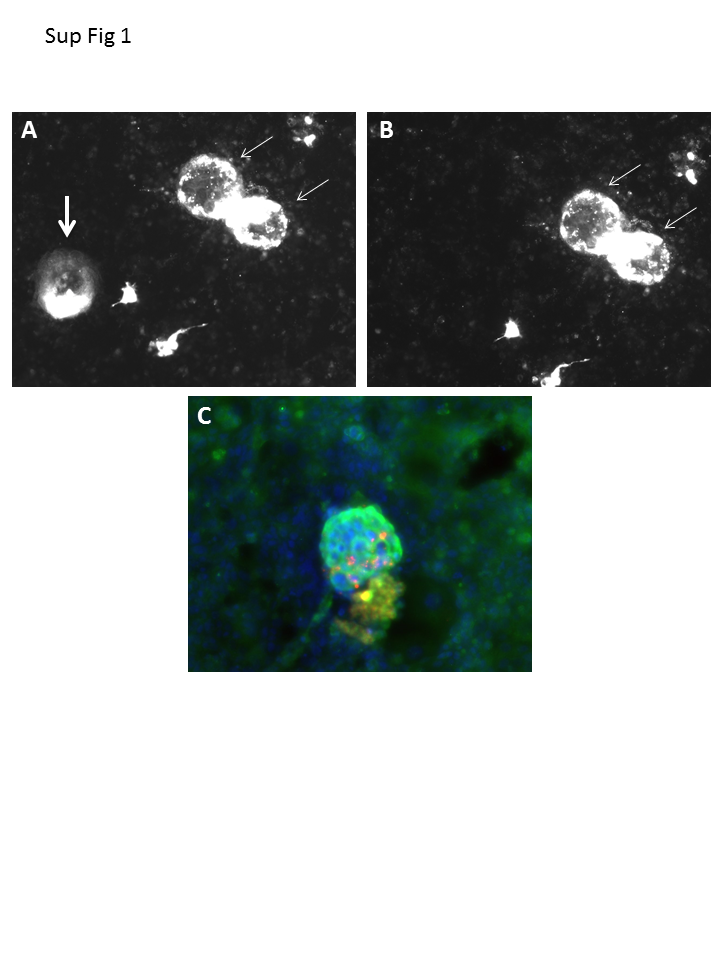

Supplement: Supplementary file 1 — Figure S1. Embryos attachment to endometrial cells. Mouse blastocysts co-incubated with endometrial cell lines for 48 h (A). Attached embryos that stayed on the plate after the plates were washed and shaken (B). Thin arrows indicate attached embryos, thick arrow indicate unattached embryos. (C) The localization of osteopentin, an adhesion molecule, between the embryo and the endometrial cells. Osteopentin-green, DAPI-blue. (TIFF 2025 kb) [file 12958_2018_329_MOESM1_ESM.tif]

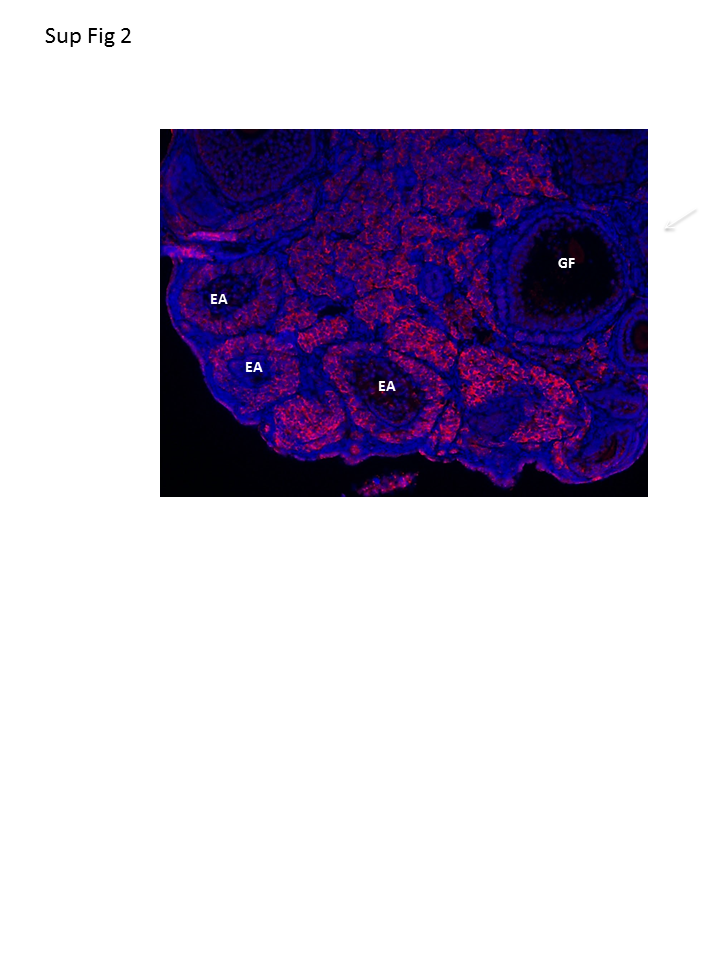

Supplement: Supplementary file 3 — Figure S2. FOXL2 localization in the ovary. FOXL2 expressed by granulosa cells of early follicles declines at later stages of folliculogenesis. Blue = DNA staining (DAPI), RED = FOXL2. EA-Early antral, GF-Graffian Folicle. (TIFF 2025 kb) [file 12958_2018_329_MOESM3_ESM.tif]

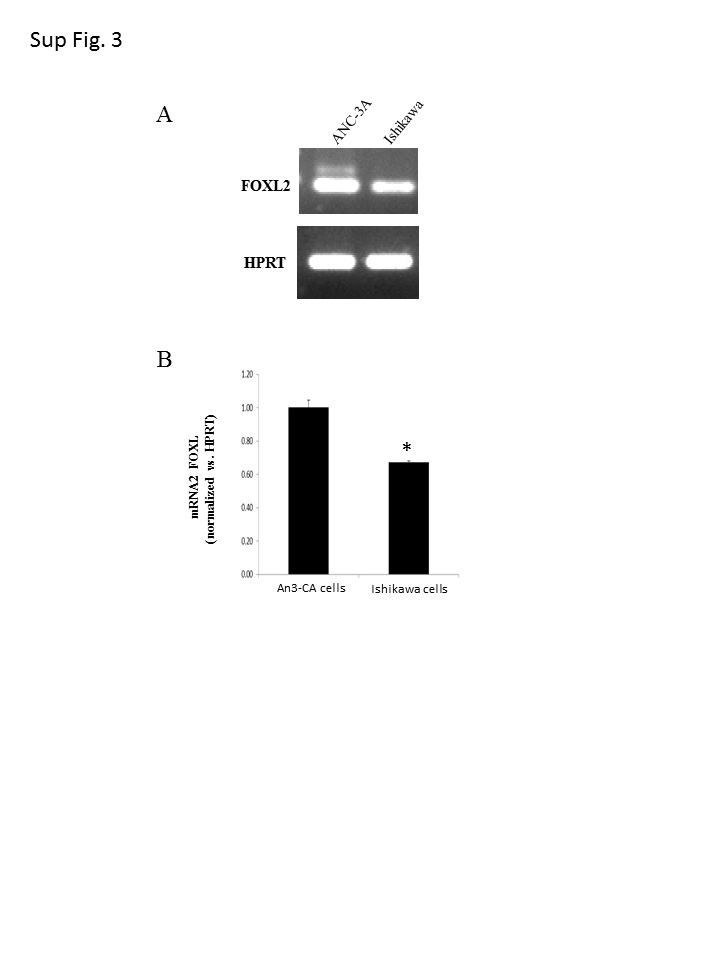

Supplement: Supplementary file 4 — Figure S3. Foxl2 expression in human endometrial cell lines. A) Human endometrial cell lines express Foxl2 mRNA. Foxl2 mRNA expression in AN3-CA and Ishikawa endometrial cell lines was analyzed by RT-PCR, using Hprt mRNA as an internal control. B) Foxl2 levels are shown as fold mRNA expression, normalized to Hprt. (TIFF 2025 kb) [file 12958_2018_329_MOESM4_ESM.tif]

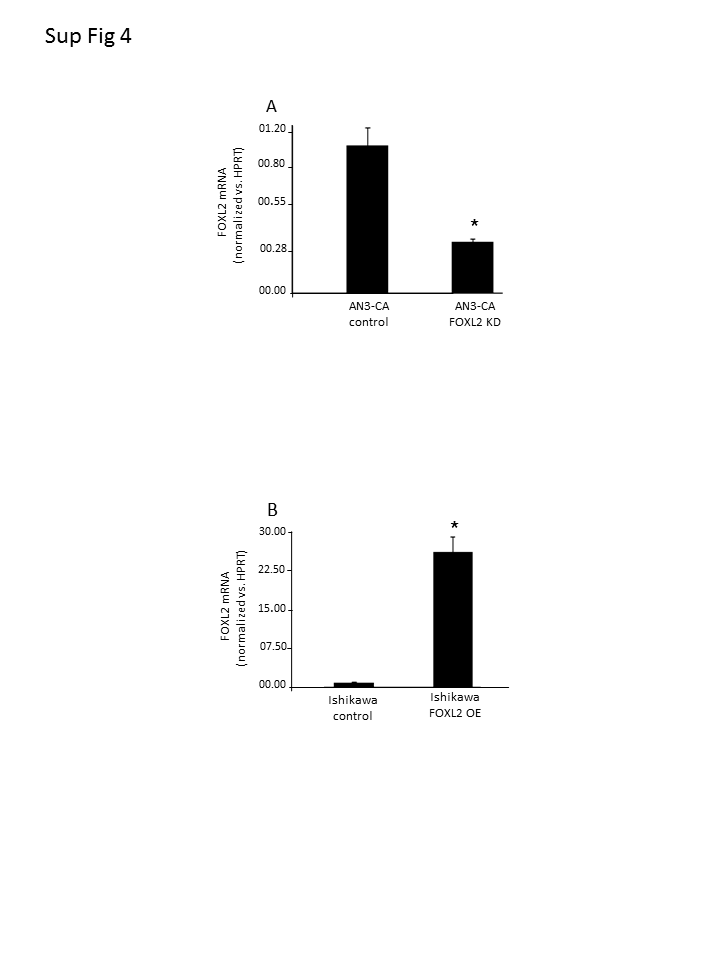

Supplement: Supplementary file 5 — Figure S4. Manipulating Foxl2 expression in human endometrial cell lines. A) Foxl2 expression is decreased in endometrial non-receptive AN3-CA cells infected with lentivirus expressing Foxl2 siRNA cassette. B) Ishikawa cells infected with lentivirus overexpress Foxl2 exhibit higher FOXL2 levels as compared to control. The results of one representative out of a total of 3 independent experiments with similar results is presented. + * p < 0.05. (TIFF 2025 kb) [file 12958_2018_329_MOESM5_ESM.tif]
