# Supplementary material for: Uterine Foxl2 regulates the adherence of the Trophectoderm cells to the endometrial epithelium
Source: Reprod Biol Endocrinol. 2018 Feb 7;16:12. doi: 10.1186/s12958-018-0329-y (PMC5804001; doi:10.1186/s12958-018-0329-y)
Supplement: Supplementary file 2 — Table S1. PCR primers list. (DOCX 15 kb) [file 12958_2018_329_MOESM2_ESM.docx]

| **Tm** | **amplicon size (bp)** | **3' sequence** | **5' sequence** | **Gene** |
| --- | --- | --- | --- | --- |
| 60 | 154 | catctggcaggaggcgta | cggggttcctcaacaactc | FOXL2 |
| 60 | 129 | gaggctgcttggttctgaag | caggcaggcagtgtatctga | FZD6 |
| 60 | 209 | ctgccgttggaagtcttgtt | ctcaagacccgctacctgtc | Wnt11 |
| 60 | 132 | gatgccctcctctgtctcag | acgcagcaacacagctacag | Kermen2 |
| 60 | 203 | agggtctgggccatagaact | gaactggcagaagaggcact | TNFIP3 |
| 60 | 165 | gggccatctggaacataaga | gaatcccagcagcagagaac | ATF3 |
| 60 | 153 | cgccgaagtctcacacagta | gaactgcggcaaagtaggag | IER3 |
| 60 | 126 | tactggccagcagttcatca | tgcagaattcctctgctcct | RGS2 |
| 60 | 187 | ctcccttctggtcagttgga | agggaattcaccccaagaac | CXCl1 |
| 60 | 101 | ggtccttttcaccagcaagct | gcagtacagccccaaaatgg | HPRT |
